# The gut microbiota of nonalcoholic fatty liver disease: current methods and their interpretation

**DOI:** 10.1007/s12072-015-9640-2

**Published:** 2015-06-12

**Authors:** Niels van Best, Peter L. Jansen, Sander S. Rensen

**Affiliations:** Department of Surgery, NUTRIM School of Nutrition and Translational Research in Metabolism, Maastricht University Medical Center, Maastricht, The Netherlands

**Keywords:** Gut microbiota, Steatosis, Steatohepatitis, Fibrosis, Cirrhosis, Bile acids

## Abstract

The role of intestinal bacteria in the pathogenesis of nonalcoholic fatty liver disease is increasingly acknowledged. Recently developed microbial profiling techniques are beginning to shed light on the nature of gut microbiota alterations in nonalcoholic fatty liver disease. In this review, we summarize the gut microbiota composition changes that have been reported during different stages of human nonalcoholic fatty liver disease, and highlight the relation between bile acids and gut bacteria in this context. In addition, we discuss the different methodologies used in microbiota analyses as well as the interpretation of microbiota data. Whereas the currently available studies have provided useful information, future large-scale prospective studies with carefully phenotyped subjects and sequential sampling will be required to demonstrate a causal role of gut microbiota changes in the etiology of nonalcoholic fatty liver disease.

## Introduction

Nonalcoholic fatty liver disease (NAFLD), the hepatic manifestation of the metabolic syndrome [1], is characterized by hepatic fat accumulation in the absence of significant alcohol consumption, viral infection, or other liver disorders [2]. NAFLD ranges from simple steatosis to inflammatory nonalcoholic steatohepatitis (NASH), with or without fibrosis. It is the most common liver disorder worldwide, and has an increasing prevalence. NASH, but not simple steatosis, frequently progresses to life threatening disorders such as cirrhosis and hepatocellular carcinoma (HCC) [3].

NAFLD pathophysiology is multifactorial, involving ecological, genetic, and metabolic factors such as limited physical activity, high energy intake, and a dysbalanced diet (e.g. too much fructose and/or saturated fat) [4]. Together with epigenetic factors, this promotes insulin resistance and hepatic fat accumulation [2, 5]. Progression towards inflammation of the steatotic liver was initially proposed to be related to endotoxemia as a result of increased gut permeability by Brun et al. [6] and Wigg et al. [7]. Subsequently, evidence accumulated that intestinal microbiota plays an important part in the pathogenesis of NAFLD [8–10]. Microbial profiling techniques developed in the past few years enabled major advances in our understanding of alterations of the gut microbiota and the role of gut bacteria in the development of NAFLD [11].

This review summarizes these recent findings, focusing on gut microbiota composition changes during the different stages of human NAFLD, and paying particular attention to the methodologies used in microbiota analyses as well as their interpretation.

## Microbiota composition in NAFLD

### Steatosis and steatohepatitis

**Fig. 1:**
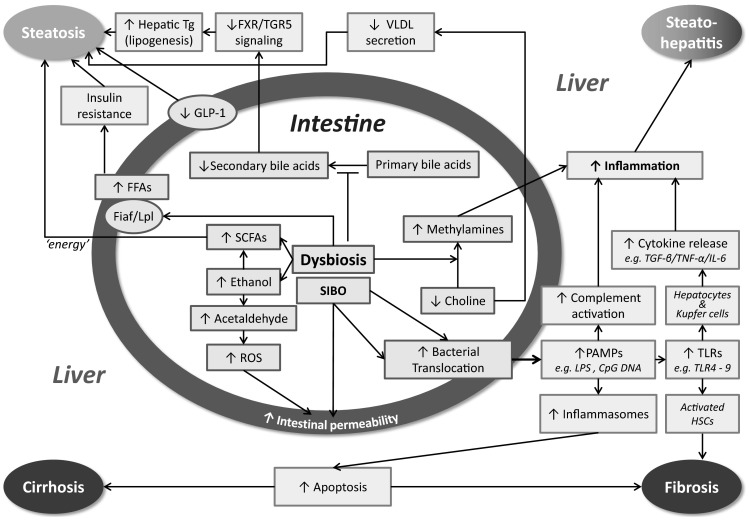
Mechanisms by which gut bacteria affect the hallmarks of nonalcoholic fatty liver disease. *SCFAs* short-chain fatty acids, *PAMPs* pathogen associated molecular patterns, *ROS* reactive oxygen species, *FFAs* free fatty acids, *Tg* triglyceride, *LPS* lipopolysaccharide, *TLR* Toll-like receptor, *SIBO* small intestinal bacterial overgrowth, *TGF*-*β* transforming growth factor-β, *IL*-*6* interleukin-6, *TNF*-*α* tumor necrosis factor-α, *HSCs* hepatic stellate cells, *Fiaf* fasting induced adipocyte factor, *Lpl* lipoprotein lipase, *VLDL* very low density lipoprotein, *GLP*-*1* glucagon-like peptide-1

There are only a limited number of studies that have examined microbiota composition in patients with simple steatosis or NASH, and these have very dissimilar results (Table 1). First of all, patients with NASH were recently shown to have a decreased abundance of bacteria belonging to the phylum Bacteroidetes compared to subjects with simple steatosis and healthy individuals as shown by qPCR [9]. In contrast, studies using sequencing techniques showed an increase of *Bacteroides*, one of the most important genera within the Bacteroidetes phylum, and a decrease of Firmicutes in NASH patients as compared to healthy subjects [8]. The lower representation of Firmicutes in NASH patients was especially due to a reduced abundance of the *Lachnospiraceae* and *Ruminococcaceae* families. However, another study demonstrated an increase of *Lachnospiraceae* and *Lactobacillaceae* in NAFLD patients, albeit without distinguishing between simple steatosis and NASH [10]. Both studies observed a decrease in members of the *Ruminococcaceae* family in NASH [8, 10]. The apparent lack of consistent changes of gut microbiota composition in NASH is further exemplified by two recent studies, showing either an overrepresentation of the genus *Escherichia* from the *Enterobacteriaceae* family in subjects with NASH [8] or no difference in *Escherichia coli* abundance in NASH patients compared to subjects with simple steatosis [9]. Despite the lack of consistent NAFLD-related gut microbiota changes, the possible overgrowth of these ethanol-producing bacteria may underlie the increased circulating ethanol levels in NASH [8]. The endogenous production of ethanol might, in turn, contribute to the formation of free fatty acids and oxidative stress (Fig. 1), further underscoring the potential role of ethanol-producing bacteria in the pathogenesis of NAFLD.

**Table 1:** Significant microbiota composition changes in nonalcoholic liver disease

Disease comparison	Samples	Microbiota variations (family_genus)	Techniques	References
Non-NASH cirrhotic patients (*n* = 181) versus NASH cirrhotic patients (*n* = 32)	Stool	↑*Bacterioidaceae* ↑*Porphyromonadaceae* ↓*Veillonellaceae*	16S rRNA MT pyrosequencing	[13]
Healthy (*n* = 17) versus NASH (*n* = 22)	Stool	↓Phylum: Bacteroidetes	qPCR	[7]
Simple steatosis (*n* = 11) versus NASH (*n* = 22)	Stool	↓Phylum: Bacteroidetes ↑ *C. coccoides*	qPCR	[7]
Healthy (*n* = 30) versus obese NAFLD (*n* = 30)	Stool	↑*Veillonellaceae* ↑*Kiloniellaceae* ↑*Pasteurellaceae* ↑*Lactobacillaceae* ↑*Lachnospiraceae* ↓*Ruminococcaceae* ↓*Porphyromonadaceae* ↑*Lactobacillaceae_Lactobacillus* ↑*Lachnospiraceae_Dorea* ↑*Lachnospiraceae_Robinsoniella* ↑*Lachnospiraceae_Roseburia* ↓*Ruminococcaceae_Oscillibacter*	16S rRNA MT pyrosequencing	[8]
Obese (*n* = 25) versus NASH (*n* = 22) *children*	Stool	↑*Enterobacteriaceae* ↑*Enterobacteriaceae_Escherichia*	16S rRNA MT pyrosequencing	[6]
Healthy (*n* = 16) versus NASH (*n* = 22) *children*	Stool	↑*Enterobacteriaceae* ↑*Enterobacteriaceae_Escherichia* ↓*Bifidobacteriaceae_Bifidobacterium* ↓*Bifidobacteriaceae* ↑*Prevotellaceae* ↑*Prevotellaceae_Prevotella* ↓*Rikenellaceae* ↓*Rikenellaceae_Alistipes* ↑*Clostridiales XI_Peptoniphilus* ↓*Lachnospiraceae* ↓*Lachnospiraceae_Blautia* ↓*Lachnospiraceae_Coprococcus* ↓*Eubacteriaceae_Eubacterium* ↓*Lachnospiraceae_Roseburia* ↓*Ruminococcaceae* ↓*Ruminococcaceae_Oscillospira* ↓*Ruminococcaceae_Ruminococcus* ↓*Ruminococcaceae_Unclassified* ↑*Alcaligenaceae*	16S rRNA MT pyrosequencing	[6]

Large scale trials, designed to identify alterations of microbiota composition in patients with simple steatosis versus NASH, are required to shed more light on the nature of the gut microbiota shifts characteristic of specific stages of NAFLD. These trials should also pay attention to the fact that most NAFLD patients are obese, since obesity itself is linked to gut microbiota composition changes, as reviewed elsewhere [12, 13]. Future studies should ideally include obese non-NAFLD patients or non-obese NAFLD patients [14] to exclude the impact of obesity, or control for obesity in statistical analyses. It should be further noticed that many studies so far excluded all taxa with an abundance below 1 %. However, even low-abundant bacteria such as *Akkermansia muciniphila* have the potential to profoundly affect host metabolism [15]. In addition, without any fundamental direct evidence provided by fecal transplantation or antibiotic studies, one cannot exclude that the described alterations in the intestinal microbiota are a consequence rather than a cause of liver disease.

### Fibrosis and cirrhosis

Surprisingly little evidence exists to date for an effect of gut microbiota on liver fibrosis. However, very recently, an elegant study was published showing that in a bile duct ligation-induced liver fibrosis model, transplantation of the gut microbiota of mice fed a high fat diet (HFD) aggravated fibrosis relative to transplanting gut microbiota of mice fed chow [16]. This was mainly attributable to an increased abundance of Gram-negative Proteobacteria; a marked decrease of *Bifidobacteriaceae* was also observed. Specific bacteria of both the Proteobacteria and Firmicutes phyla produce enzymes catalyzing choline conversion into methylamines [17]. The latter may promote liver inflammation via the portal vein, and decreased choline levels have already been associated with fibrosis progression [18]. However, future studies should ascertain to what extent the described microbiota composition changes in fibrosis affect choline metabolism.

Cirrhosis patients often have a higher proportion of potentially pathogenic bacteria and a reduction of autochthonous (resident) bacteria compared to healthy individuals (Table 2) [19–21]. Common differences include a decrease in families with the potential to convert primary into secondary bile salts (*Lachnospiraceae* and *Ruminococcaceae*) in cirrhosis, and overgrowth of the Gram-negative *Enterobacteriaceae* family, similar to what is observed in NASH [22]. This suggests that bile salts and endotoxin [23] may play a role in the pathogenesis of cirrhosis. Additional gut microbiota changes in patients with cirrhosis include a decrease in *Clostridiales XIV* and an increase in *Enterococcaceae* and *Staphylococcaeae* [19] as well as overgrowth of *Veillonellaceae* [21].

**Table 2:** Significant microbiota composition changes in cirrhosis

Disease comparison	Samples	Microbiota variations (family_genus)	Techniques	References
Healthy (*n* = 25) versus cirrhotic compensated outpatients (*n* = 121), cirrhotic decompensated outpatients (*n* = 54), cirrhotic inpatients (*n* = 44)	Stool	↓*Clostridiales XIV* ↓*Ruminococcaceae* ↓*Lachnospiraceae* ↑*Enterococcaeae* ↑*Staphylococcaceae* ↑*Enterobacteriaceae*	16S rRNA MT Pyrosequencing	[13]
Healthy (*n* = 17) versus liver cirrhosis (*n* = 36)	Sigmoid mucosa	↑*Burkholderiaceae_Burkholderia* ↑*Burkholderiaceae_Ralstonia* ↑*Clostridiaceae_Clostridium* ↑*Clostridiaceae_other* ↑*Enterobacteriaceae_Proteus* ↑*Enterococcaceae_Enterococcus* ↓*Incertae Sedis XIV_other* ↓*Lachnospiraceae_Dorea* ↓*Lachnospiraceae_unclassified* ↓*Ruminococcaceae_Subdoligranulum* ↓*Veillonellaceae_Acidaminococcus*	16S rRNA MT Pyrosequencing	[14]
Healthy (*n* = 14) versus liver cirrhosis (*n* = 47)	Stool	↑*Enterobacteriaceae* ↑*Veillonellaceae* ↓*Lachonospiraceae* ↓*Ruminococcaceae* ↓*Ruminococcaceae_Blautia*	16S rRNA MT Pyrosequencing	[15]
Healthy (*n* = 4) versus liver cirrhosis (*n* = 6)		↑*Enterobacteriaceae* ↑*Enterococcus*	RT-PCR	[16]
Healthy (*n* = 98) versus liver cirrhosis (*n* = 83)	Stool	↓*Bacteroidaceae _Bacteroides* ↓*Eubacteriaceae _Eubacterium* ↓*Rikenellaceae _Alistipes* ↑*Veillonellaceae _Veillonella* ↑*Streptococcaceae _Streptococcus* ↑*Clostridiaceae_Clostridium* ↑*Prevotellaceae _Prevotella*	Illumina	[18]

Another cohort of cirrhotic patients revealed a decrease of the genera *Bacteroides*, *Eubacterium*, and *Alistipes*, whereas *Clostridium* and *Prevotella* were increased compared to healthy controls [24]. However, the most abundantly enriched species in these cirrhotic patients belonged to the *Streptococcus* and *Veillonella* genera. Remarkably, these genera comprise oral species that might invade the gut and contribute to small-intestinal bacterial overgrowth, which frequently occurs in NASH and cirrhosis [7, 25]. Furthermore, the lower microbial richness and reduced abundance of butyrate-producing species with anti-inflammatory properties (*F. prausnitzii*, *Coprococcus comes*, *Lachnospiraceae* spp., *Ruminoccaceae* spp.) suggest that patients with cirrhosis have a less “healthy” microbiota [24, 26, 27].

Cirrhotic patients with NASH further demonstrated a decrease in *Veillellaceae* and an increase in *Bacteroidaceae* and *Porphyromonadaceae* families compared to cirrhotic patients without NASH [19]. Although the abundance of *Enterobacteriaceae* was increased in NASH patients compared to healthy individuals, *Enterobacteriaceae* in NASH patients within a cirrhotic cohort were not affected. The latter might be due to the high proportion of *Enterobacteriaceae* in cirrhotic patients [19, 21, 22]. Interestingly, Bajaj and colleagues showed that the microbiome in cirrhotic patients with stable disease remains unaltered over time, suggesting that the composition of the microbiome can be used as a potential disease marker [19]. All in all, it appears that different stages of NAFLD are associated with different microbiota compositions, although these need to be better defined.

## Gut microbiota, bile acids, and hallmarks of NAFLD

The mechanisms by which gut bacteria affect the various manifestations of NAFLD have been reviewed recently by Schnabl et al. [28]. The most relevant insights obtained in this burgeoning field of research are summarized in Fig. 1. In view of the accumulating evidence for the role of bile acids in the treatment of NAFLD [29], we will here focus on the intimate and reciprocal relation between bile acids and the gut microbiota.

Bacteria are needed for deconjugation, 7α-dehydroxylation, and dehydrogenation of primary bile acids. Furthermore, the conversion of primary to secondary bile acids entirely depends on bacteria. Interestingly, germ-free mice have an increased bile acid synthesis in parallel with a decreased fecal bile acid output and an expanded circulating bile acid pool [30]. Thus, there appears to be a relation between the gut microbiota, bile acid synthesis in the liver, and bile acid uptake in the terminal ileum. Fibroblast growth factor 19 (FGF19; Fgf15 in rodents) plays a major role in this by linking events in the gut to metabolism in the liver [31, 32]. Upon activation of the Farnesoid X receptor (FXR) by bile acids, FGF19 is produced in the ileum and secreted into the portal circulation. In the liver, FGF19 action ultimately results in reduced transcription of Cyp7A1, the rate-limiting enzyme for bile acid synthesis [31].

Recent experiments illustrate the interdependence of gut microbiota and hepatic bile acid synthesis. Bile acids chenodeoxycholate (CDCA) and cholate (CA) act as FXR agonists, while tauro-α-muricholic acid (TαMCA) and tauro-β-muricholic acid (TβMCA) antagonize FXR [30, 33]. In germ-free mice, TβMCA is produced relatively in excess over CA, suppressing the generation of Fgf15 thereby increasing primary bile acid synthesis [30], with TβMCA in excess over CA. In conventional mice, the TβMA/CA ratio is more in favor of CA, limiting bile acid synthesis. This has implications for the actions of antibiotics. The administration of ampicillin to mice decreases Fgf15, thereby increasing Cyp7a1 expression and the synthesis of primary bile acids [30, 34]. Miyata et al. [35] explain the reduced Fgf15 expression by a lack of secondary bile acids in antibiotic-treated mice. A more likely explanation is that the TβMCA/CA ratio increases under antibiotics, causing decreased Fgf15 expression and increased bile acid synthesis [30]. Of note, humans do not produce TαMCA and TβMCA and therefore the effect of antibiotics on bile acid synthesis in humans may not be the same as in mice.

Bile acids have a direct antimicrobial effect and affect microbiota composition [36, 37]. For example, administration of CA to rats induced outgrowth of bacteria in, for example, the genus of *Clostridia*. These efficiently transform primary bile acids into deoxycholic acid (DCA) by 7α-dehydroxylation [37]. These changes are similar to the changes seen with a high fat diet. Furthermore, bile duct ligation in mice induces bacterial overgrowth, mucosal injury, and bacterial translocation [38, 39]. Lack of FXR-mediated production of bacteriostatic angogenin1 may play a role in this, but the details need to be elucidated. Patients with advanced cirrhosis have a reduced fecal concentration of total bile acids and a predominance of primary bile acids [21]. Advanced cirrhotics also have a higher abundance of *Enterobacteriaceae* and a decrease of *Clostridia* [19]. These intestinal bile acid alterations may contribute to changes of the microbiota. One can argue that in cirrhotics with a contracted bile acid pool, FXR activation in the ileum will be reduced. This leads to upregulation of the apical sodium-dependent bile salt transporter in the terminal ileum, reducing spill-over of bile acids from ileum to cecum. Bile acids in the cecum have a strong effect on 7α-dehydroxylating *Clostridia* [21]. Reduction of the cecal/colonic bile acid concentration therefore decreases conversion of primary bile acids into DCA and LCA (lithocholic acid).

In contrast, NAFLD-inducing high fat diets (HFD) in mice increase the conversion of primary to secondary bile acids. DCA has pro-inflammatory and DNA-damaging properties. Yoshimoto et al. [40] report that DCA induces a ‘senescence-associated secretory phenotype’ in hepatic stellate cells. Among the secretory products are IL-6 and PAI-1, factors known to induce cancer and obesity [41]. However, diet-induced obesity by itself is not enough to induce hepatocellular cancer (HCC) in mice; it was the combination of HFD and treatment with a carcinogen that was required. The role of DCA in this model was shown by administration of an inhibitor of 7α-dehydroxylation, which decreased serum DCA concentration and HCC development. In contrast, adding DCA to the HFD increased HCC development [40], the ultimate consequence of NAFLD.

From these studies it is clear that bile acids are major players in the interaction between the gut microbiota and the host. Bile acids affect signaling paths, not only those mediated by FXR and FGF19, but also pathways regulated by the xenobiotic receptor PXR, the vitamin D receptor VDR, the G protein-coupled transmembrane receptor TGR, the muscarinic receptor, and the conjugated bile acid receptor [42]. These receptors affect metabolism in a wide variety of cells and organs both within and outside the enterohepatic circulation. For drug development, this new knowledge provides opportunities and challenges. Little attention has been paid yet to the effect of new potent FXR agonists like obeticholic acid on the microbiota. In view of the effects these drugs have on bile acid metabolism and FGF19 expression, they likely will also affect the microbiota. Furthermore, bile acid-mediated activation of TGR5 induces secretion of the glucose homeostasis regulating hormone glucagon-like peptide-1 (GLP-1) [43], which levels are decreased in NAFLD patients [44]. Other microbial metabolites such as indole and butyrate are also able to promote GLP-1 secretion [45, 46], and modulation of the gut microbiota by prebiotics or antibiotics affects GLP-1 secretion as well [46–48]. This further underscores the close interaction between the gut microbiota, bile acids, and gut hormones involved in metabolism.

## How to study and interpret microbiota

To interpret the relevance of published data on microbiota in NAFLD, both the characterization of the disease and the type of microbial assays employed need to be taken into account. Importantly, the intestinal microbiota composition can be affected by even small changes in experimental methods at several steps from sample collection to statistical analysis, resulting in a different outcome of apparently identical studies. The most crucial technical and analytical aspects of microbial profiling, i.e. factors that may influence the results and/or result in possible bias, will be discussed here.

### Sampling and storage

It is essential that sampling and storage methods do not modify microbiota composition by themselves. An important consideration in this respect concerns the type of samples collected, which are commonly stool or endoscopic biopsies. Stool samples are non-invasive and easy to obtain. However, the colonic *mucosal* microbiota has been shown to deviate considerably from *stool* microbiota, also in cirrhotic patients [49–51]. Moreover, whereas microbiota composition along the colon was considered homogenous only a decade ago [50, 51], recent high-throughput microbial profiling techniques revealed that the mucosal microbiota varies along the length of the gut [52, 53].

Immediate freezing of microbiota-containing samples is regarded the gold standard for long-term storage. However, this is a logistic challenge in large cohort studies which usually rely on fecal swabs [54]. Fecal swabs are commonly stored for short-term at room temperature in specific media in which obligate anaerobes can survive [55]. Recently, the impact of different sampling (fecal aliquots and fecal swabs) and storage techniques (−80, −20, +4 °C for 1 week and RT for 24 h) on fecal microbiota composition was examined in healthy and diseased individuals [56]. Although no significant effect of storage temperature on microbiota composition was observed during transport (24 h), fecal swabs stored in Cary-Blair medium showed an enrichment of *Ruminococcus* and *Enterobacteriaceae* in comparison to fecal aliquots stored at −80 °C. Therefore, it is recommended to use a single uniform method within one study to minimize possible bias. Additionally, it is crucial to only compare results that have been obtained with the same sample methodology and from the same type of sample.

As for the design of studies, large-scale well-defined prospective cohorts of patients that have been carefully phenotyped are essential. In these studies, factors known to influence gut microbiota composition should be taken into account. In particular, diet and use of antibiotics, probiotics, and prebiotics should be well-documented. Furthermore, sex-specific differences in the human colonic microbiota have recently been shown [52] and should be considered when setting up microbiota studies.

### Microbial screening techniques

The human gut mainly harbors strictly anaerobic microbial species that are difficult to culture. Therefore, several culture-independent techniques have been introduced in recent decades to analyze composition and complexity of the intestinal microbiota. These techniques include (quantitative) polymerase chain reaction [(q)PCR], PCR followed by denaturing gradient gel electrophoresis, fluorescent in situ hybridization (FISH), and DNA microarrays that hybridize ribosomal RNA (rRNA) sequences with probes.

Although these methods are useful for rapid microbial profiling, they usually do not provide detailed taxonomic data. For that reason, the most important techniques nowadays are high-throughput next-generation sequencing followed by bioinformatics analysis. These sequence methods are commonly based on analysis of the 16S rRNA gene which is present in all bacteria and archaea, consisting of nine unique hypervariable regions (V1–V9) and bordered by highly conserved regions [57]. Despite the fact that other genes have been suggested, sequencing of 16S rRNA remains the gold standard to analyze the microbiota in view of the completeness of reference databases, low costs, and advanced bioinformatics software available. Different next-generation sequencers can be used to analyze the 16S rRNA gene. However, the majority utilizes either Illumina sequencing (Illumina Inc., San Diego, CA) or 454 pyrosequencing (Roche, Brandfort, CT). These widely popular approaches have distinct coverage ratios, sequence lengths, and construction mechanisms. 454 pyrosequencing generates longer sequences which increases taxonomic accuracy, whereas Illumina provides higher coverage at lower cost per sample [58]. It should be noted that the selection of primers, the number of sequences, costs, and aim of the experiment are strongly interdependent. Therefore, it is essential to balance these factors to achieve the optimum amount of information.

Although 16S sequencing is the most commonly used technology in microbiome studies, it is important to understand its limitations. Differences in DNA extraction methods, PCR errors, and discrepancy in 16S gene copy numbers all affect the proportions of bacteria detected [59–61]. The latter leads to bias in the detection of unknown or unclassified bacteria (dead or alive) and taxonomic characterization. Moreover, both 454 pyrosequencing and Illumina display sequencing error rates; pyrosequencing is associated with relatively more insertions and deletions, whereas Illumina has more mismatches [62, 63]. Therefore, it is crucial to consider standardized control sequences to estimate the exact error rate for each experiment. Additionally, despite the fact that sequencing of 16S rRNA provides insight into overall microbiota composition, it does not provide data on their functions and interactions. Finally, it should be noted that it was recently found that in certain conditions, next-generation sequencing data were found to be less representative of the ‘real’ microbiota than culture-based methods [64].

### Metagenomics and metabolomics

As opposed to sequencing of marker genes such as the 16S rRNA gene, sequencing of the entire genomic content of microorganisms (the ‘microbiome’) provides more specific information on their potential functional roles. These metagenomic or metatranscriptomic analyses, referred to as shotgun sequencing, are especially appropriate for linking microbial communities with functional potential and activity in the human gut. In addition, metabolomics—the quantitative assessment of metabolic responses of organisms to genetic or pathophysiological changes—is a powerful approach and also key to unraveling specific host-microbe interactions. Metagenomic approaches relate the microbiome to phenotype changes in disease, whereas metabolomic approaches relate the metabolic profile with disease phenotypes [65]. The latter analyses are principally based on proton nuclear magnetic resonance (1H-NMR) spectroscopy and mass spectrometry methods. Ideally, these analytical techniques should be complemented for the most detailed characterization of microbial metabolites obtained from feces, blood, urine, or intestinal tissues. In contrast to just sequencing the microbiota, shotgun-sequencing and metabolomics require direct freezing of fecal samples.

### Analysis with bioinformatics

16S rRNA gene sequences can be analyzed with several tools, especially with QIIME [66] and Mothur software [67], which are reviewed in detail elsewhere [65, 68, 69]. These approaches produce phylogenetic trees and assessments of bacterial diversity within samples (α-diversity) and between samples (β-diversity). To this end, sequences are clustered into taxonomic groups, referred to as operational taxonomic units (OTUs), by comparing them across samples (de novo) or to reference data such as Greengenes [70]. Importantly, a reference-based approach provides a more straightforward interpretation and enables the comparison of data with different sequenced regions of the 16S rRNA gene. However, a crucial consideration and limitation of this technique is the common existence of unclassified bacteria in these databases and the complex taxonomic clustering including discrimination of related bacterial types. To reduce the effect of sequencing errors, it is recommended to disable the formation of new clusters with sequences that were not detected in any reference database. Fortunately, the increasing developments in the field of microbiota contribute to a high rate of classifying and discovering novel bacteria species.

## Conclusion and perspectives

Our progressive knowledge of the role of the gut microbiota in NAFLD is rapidly expanding due to improved DNA sequencing techniques. However, currently available studies show a marked discrepancy in results which is likely related to small sample size, variability in patient cohorts, and limited phenotyping of liver disease. Different sampling techniques and analysis methods likely also underlie the lack of consistent data. The key challenge now is to execute large-scale prospective studies with carefully phenotyped subjects; sequential samples should be obtained to demonstrate causality of gut microbiota changes in the etiology of NAFLD/NASH. In addition, diet, medicine use, and sampling methods should be well documented and considered before drawing conclusions. Importantly, metatranscriptomic and metabolic approaches are lacking and are urgently needed to assess the specific functional role of a certain microbial community. Providing insight into these aspects will help in understanding NAFLD pathophysiology and might eventually yield non-invasive biomarkers. However, next-generation sequencing techniques are currently not able to characterize the entire microbiota and their reproducibility has to be increased to use the microbiome as a diagnostic biomarker. Novel molecular methods, such as the promising IS-pro technique, may contribute to this [71]. Despite these challenges and the fact that the progression of NAFLD relies on multiple hits, the intestinal microbiota appears to represent an important factor that contributes to several aspects of NAFLD, and should be considered in any future mechanistic study.
